# Suppressing of slow magnetic relaxation in tetracoordinate Co(II) field-induced single-molecule magnet in hybrid material with ferromagnetic barium ferrite

**DOI:** 10.1038/srep10761

**Published:** 2015-06-03

**Authors:** Ivan Nemec, Radovan Herchel, Zdeněk Trávníček

**Affiliations:** 1 Department of Inorganic Chemistry, Regional Centre of Advanced Technologies and Materials, Faculty of Science, Palacký University, 17. listopadu 12, CZ-771 46 Olomouc, Czech Republic

## Abstract

The novel field-induced single-molecule magnet based on a tetracoordinate mononuclear heteroleptic Co(II) complex involving two heterocyclic benzimidazole (bzi) and two thiocyanido ligands, [Co(bzi)_2_(NSC)_2_], (CoL4), was prepared and thoroughly characterized. The analysis of AC susceptibility data resulted in the spin reversal energy barrier *U* = 14.7 cm^−1^, which is in good agreement with theoretical prediction, *U*_theor._ = 20.2 cm^−1^, based on axial zero-field splitting parameter *D* = −10.1 cm^−1^ fitted from DC magnetic data. Furthermore, mutual interactions between CoL4 and ferromagnetic barium ferrite BaFe_12_O_19_ (BaFeO) in hybrid materials resulted in suppressing of slow relaxation of magnetization in CoL4 for 1:2, 1:1 and 2:1 mass ratios of CoL4 and BaFeO despite the lack of strong magnetic interactions between two magnetic phases.

In recent two decades, there have been a lot of efforts invested in research of magnetic anisotropy of high spin first-row transition metal mononuclear complexes^1^. The research interest was accentuated by the ability of such complexes to serve as good models of local magnetic behavior on centers in polynuclear complexes, which were subject of magneto-chemical interest initially. This was due to the phenomenon of a slow-relaxation of magnetization (SRM) which allows individual complex molecules to behave as molecular nanomagnets, so called single –molecule magnets (SMMs) and this was discovered on polynuclear species firstly^2^. The main characteristic of SMMs is that they retain their magnetic moment (magnetic dipole orientation) even after the removal of external magnetic field. Such behavior can be observed due to an existence of energy barrier acting against thermally induced spin reversal and the height of energy barrier is defined as *U* = |*D*|(*S*^2^ – 1/4), where *S* is the half-integer spin of the metal center ground state and *D* is the axial parameter of zero-field splitting (ZFS), while the negative value of *D* parameter assures generation of the aforementioned barrier. Nevertheless, it must be noted that SMMs with the positive value of *D* parameter accompanied by large rhombic anisotropy (*E*) were reported previously^3^. Furthermore, when a quantum tunneling between the *M*_S_ and *M*_−S_ states is too fast the small external field can suppress this process and SRM can be observed by using AC susceptometry measurements in non-zero static magnetic field and this kind of compounds is named as field-induced single-molecule magnets. The magnetic anisotropy research has become increasingly important when novel class of mononuclear SMMs (so called single-ion magnets, further abbreviated as SIMs) emerged. First reports dates back to 2003 when Ishikawa and coworkers reported on first Dy and Tb phthalocyanine double-decker complexes exhibiting SRM^4^. The first evidence of the first-row transition metal mononuclear complex exhibiting SRM was reported in 2010 on a trigonal pyramidal complex of Fe(II) by Long and coworkers^5^. The discovery of the first Co(II) SIM followed next year, in 2011, T. Jurca *et al*. reported on SRM in pentacoordinate mononuclear Co(II) compounds with isothiocyanido and bis(imino)pyridine pincer ligands^6^. Further reports on Co(II) SIMs aim dominantly on low-coordinate species, such as tridentate^7^, tetracoordinate^8^, pentacoordinate^9^, but also hexacoordinate SIM complexes were reported^10^. The relatively frequent occurrence of the SIM phenomenon in Co(II) compounds motivated us to study magnetic anisotropy of tetracoordinate Co(II) compounds. These are synthetically easily available and usually sufficiently air-stable and therefore, such compounds are appropriate candidates for extensive and advanced studies.

In this work, we report on synthesis, crystal structure and magnetic properties of mononuclear tetracoordinate complex [Co^II^(bzi)_2_(NCS)_2_] (CoL4), where bzi = benzimidazole. In order to prove field-induced SRM in CoL4 the thorough study of magnetic properties was done by analysis of experimental data acquired by DC and AC magnetometry. Additionally, the experimentally obtained results were compared with those acquired using *ab initio* (DFT and CASSCF) calculations. Moreover, inspired by our recent research involving a study of interactions between molecule-based metamagnet {[Ni(en)_2_]_3_[Fe(CN)_6_]_2_⋅3H_2_O}_*n*_^11^ and nanocrystalline magnetite Fe_3_O_4_ resulting in magnetic superstructure^12^, we decided to investigate the impact of ferromagnetic barium ferrite BaFe_12_O_19_ (BaFeO) upon herein reported static and dynamic magnetic properties of a single-ion molecule magnet CoL4. Therefore, the heterogeneous solid state mixtures of CoL4 and BaFeO in mass ratios 1:2, 1:1 and 2:1, respectively, were prepared by ball milling and characterized by DC magnetization and AC susceptibility measurements with the aim to answer remarkable questions regarding the possibility of dipolar/exchange magnetic interactions between two magnetic components and possible influence of ferromagnetic component on SRM of CoL4.

## Results and Discussion

### Synthesis and X-ray structure analysis

The synthesis of CoL4 is very straightforward and facile: CoCl_2_ was mixed with KNCS in 1:2 molar ratio in methanol, producing characteristic cobalt-blue colored solution. Then, the stoichiometric amount of bzi was added during stirring. The solution was further stirred under heating for 15 minutes and then was filtered off through the paper filter. The dark blue single crystals appeared after 3 days of slow evaporation of mother liquor.

The crystal structure of CoL4 was determined by a single crystal X-ray diffraction analysis. The compound crystallizes in triclinic space group *P*-1. The molecular structure of CoL4 consists of two bzi and NCS^−^ ligands coordinated to Co(II) center through nitrogen atoms, thus forming a pseudotetrahedral coordination polyhedron, with the {CoN_2_N’_2_} chromophore. The Co–N bond lengths are a bit shorter in the case of NCS^−^ ligands as compared to bzi ones (in Å): *d*(Co–N_NCS_) = 1.933(5), 1.948(4), *d*(Co–N_bzi_) = 1.988(3), 1.993(4). The angular distortion of the coordination polyhedron is more obvious while the chromophore angles differ from the ideal tetrahedral angle (*α*_Td_ = 109.5°). Such distortion can be described by previously defined parameter *δ*^13^ which includes the deviation of a sum of the angles *α* = N_NCS_–Co– N_NCS_ and *β* = N_bzi_–Co–N_bzi_ from the pair of the ideal tetrahedral angles: *δ* = 2·*α*_Td_ – (*α* + *β*). The *δ* parameter adopts relatively large negative value of −6.3°. However, there is another way how to define total angular distortion by sum of the deviations from ideal *α*_Td_ calculated for all chromophore angles *γ*_i_: *∆* = Σ_i_|*γ*_i_−*α*_Td_|. and *∆*(CoL4) = 22.3°. Summary of the selected structural parameters for CoL4 and other previously published tetracoordinate [Co(L)_2_(A)_2_] compounds^14^,^15^,^16^,^17^ are given in Table 1 (L1 = a monodentate ligand, A = an anionic ligand).

**Figure 1:**
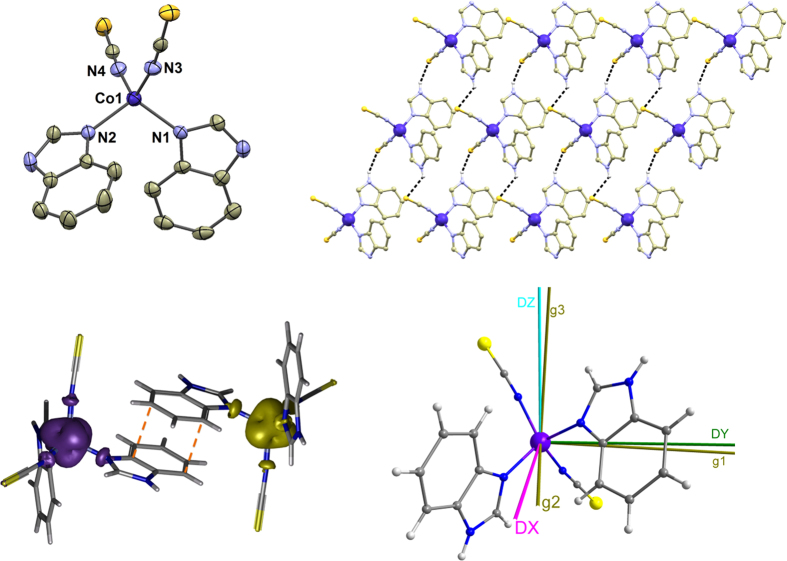
Top Left: Molecular structure of CoL4. Hydrogen atoms are omitted for clarity. Selected bond lengths and angles (in Å and °): Co1–N4 = 1.933(4), Co1–N3 = 1.947(4), Co1–N1 = 1.988(3), Co1–N2 = 1.993(3), N4–Co1–N3 = 115.69(15), N–Co1–N1 = 101.95(15), N3–Co1–N1 = 110.34(14), N4–Co1–N2 = 113.09(15), N3–Co1–N2 = 106.16(15), N1–Co1–N2 = 109.55(14). *Top Right*: Fragment of the crystal structure of CoL4. The N–H···S non-covalent contacts are displayed as black dashed lines. Hydrogen atoms are omitted for clarity, except for those involved in non-covalent contacts. *Bottom Left:* A perspective view on the interaction of aromatic rings in CoL4 with the highlighted shortest C···C distances (dashed lines), showing calculated spin density distribution using B3LYP/def2-TZVP(-f) for the broken symmetry spin state. Positive and negative spin densities are represented by violet, and yellow surfaces, respectively. The isodensity surfaces are plotted with the cutoff values of 0.005 ea_0_^−3^. *Bottom Right*: The CASSCF/NEVPT2 calculated principal axes of ZFS D-tensor labeled DX, DY, DZ and axes of *g*-tensor labeled as *g*1, *g*2, *g*3 visualized together with molecular structures of CoL4.

The crystal structure of CoL4 is stabilized by several weak non-covalent contacts. Firstly, the 2D sheet of the [Co(bzi)_2_(NCS)_2_] molecules is formed due to the weak N-H···S contacts (Fig. 1). Each complex molecule acts simultaneously as a donor and acceptor of two symmetrically independent contacts with *d*(N···S) = 3.369(3) and 3.353(3) Å. Secondly, the supramolecular [Co(bzi)_2_(NCS)_2_] ···[Co(bzi)_2_(NCS)_2_] dimers are formed in the crystal structure due to the interactions of the aromatic rings (Fig. 1). The hydrogen bonding and *π*–*π* interactions are well-known mediators of the magnetic exchange interactions, therefore, in the case of CoL4 it can be doubt if the system is magnetically isolated. However, the lengths of the possible super-exchange pathways through the N-H···S contacts are too long (based on the lengths of non-covalent contacts, *vide infra*, and Co···Co distances of involved complex molecules, 9.3540(9) and 9.5037(9) Å) and no directly coordinated atoms are involved in this kind of very weak hydrogen bonding^18^. The present *π*–*π* stacking is also quite distant with the shortest C···C distances equaled to 3.354 (5) Å (Fig. 1). On the other hand, it must be noted that such C···C distance might be indication of the non-covalent magnetic exchange pathway mediated by *π*–*π* stacking interaction^19^.

### Ab initio calculations

Theoretical calculations using the ORCA computational package were performed in order to: *i*) confirm that CoL4 molecules are magnetically isolated and *ii*) predict magnetic anisotropy of this tetracoordinate complex to help with forthcoming magnetic analysis. Firstly, DFT calculations of the isotropic exchange constant *J* in molecular dimeric fragment {[Co(bzi)_2_(NSC)_2_]}_2_ of CoL4 (Fig. 1) were performed with the aim to confirm/exclude possible magnetic interactions through π-π stacking, because very recently a Co(II) monomeric system was reported, where this type of non-covalent interaction (C···C distance *ca*. 3.37 Å) resulted in a ferromagnetic exchange (*J* = + 1.4 cm^−1^)^20^. Well established B3LYP functional was utilized to calculate energy difference Δ between the high spin (HS) and broken-symmetry (BS) spin states


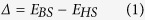


where the following spin Hamiltonian for a dimer was used


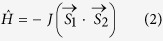


Then, the isotropic exchange J-values were calculated by Ruiz’s approach^21^,^22^ as


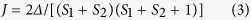


which resulted in a negligible value of *J* equaled to -0.01 cm^−1^. The spin densities of the BS spin calculation are depicted in Fig. 1.

**Figure 2:**
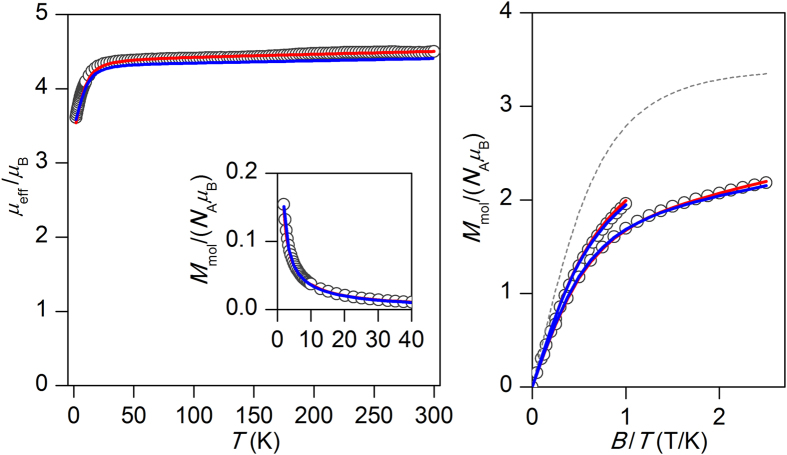
The magnetic data for CoL4. *Left*: the temperature dependence of the effective magnetic moment and molar magnetization measured at *B* = 0.1 T. *Right*: the reduced magnetization data measured at *T* = 2 and 5 K. Empty circles – experimental data, red full lines – calculated data using the equation 5, with *g* = 2.27, *D* = –10.1 cm^−1^ and *χ*_TIP _= 5.3⋅10^−9^ m^3^mol^−1^, dashed line – the calculated Brillouin’s function for *S* = 3/2 and *g* = 2.27, blue full lines – calculated data using the CASSCF/NEVPT2 energy levels.

Additionally, we performed *ab initio* calculations of ZFS parameters based on state average complete active space self-consistent field (SA-CASSCF) wave functions complemented by N-electron valence second order perturbation theory (NEVPT2) with the active space defined as CAS(7,5). The resulting values of ZFS parameters were as follows: *D* = −11.1 cm^−1^ and *E*/*D* = 0.050. Negative *D*-parameter is in agreement with the magneto-structural correlation presented in Table 1. In addition, *g*-tensor values were found as: *g*_1_ = 2.173, *g*_2 _= 2.182, *g*_3 _= 2.311 resulting in *g*_iso_ = 2.222. Both *g*-tensor and *D*-tensor almost coincide as can be seen in Fig. 1, so we can conclude from their mutual relationship that *g*_x_ = *g*_2_, *g*_y_ = *g*_1_, and *g*_z_ = *g*_3_. In next step, we utilized the respective *ab initio* CASSCF/NEVPT2 spin-orbit coupling, orbital and spin angular momentum matrices


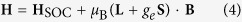


to calculate all 120 energy levels for any orientation of magnetic field **B**_*a*_, followed by integral calculation of both temperature and field dependent magnetization data, which are in good agreement with the experimental ones (Fig. 2). The same quality of agreement between theory and experiment we achieved for a hexacoordinate Co(II) field-induce single-ion magnet^23^, thus showing that this theoretical protocol is suitable for study of Co(II) complexes in relationship to their ZFS tensor and magnetic properties.

### Static magnetic properties of CoL4

Temperature and field dependent magnetic data of CoL4 are depicted in Fig. 2. The effective magnetic moment *μ*_eff_ starts at the value of 4.50 *μ*_B_ at room temperature, which is considerably higher than the spin-only value for the *S* = 3/2 and *g* = 2.0 (*μ*_eff_/*μ*_B _= 3.87) as a result of significant contribution of the orbital angular momentum to the ground state. The *μ*_eff_/*μ*_B_ starts to decrease on cooling below 30 K reaching the value of 3.62 at *T* = 1.9 K, which is caused by the zero-field splitting (ZFS). The same phenomenon is also responsible for the large deviation of the isothermal magnetization curves measured at *T* = 2 and 5 K up to 5 T from the theoretical Brillouin’s function (Fig. 2). As a result of DFT calculations, we can consider Co(II) atoms magnetically well separated from each other and we can safely use the spin Hamiltonian formalism for a monomeric complex to interpret experimental data of CoL4 as follows^24^


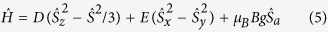


where *D* and *E* are the single-ion axial, and rhombic ZFS parameters, respectively, and *a* defines orientation of the magnetic field vector, **B**_*a*_ = *B*(sin*θ*cos*ϕ*, sin*θ*sin*ϕ*, cos*θ*). The final calculated molar magnetization was calculated as an integral average in order to properly simulate powder sample signal, using FORTRAN subroutine QROMB^25^.


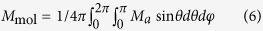


Both temperature and field dependent magnetization data were fitted simultaneously with the aim to obtain plausible parameters and it was found that satisfying fit was already obtained when rhombic anisotropy was neglected. As a result, the best-fitted parameters were found as *g* = 2.27, *D* = –10.1 cm^−1^ and *χ*_TIP_ = 5.3 ⋅10^−9^ m^3^mol^−1^ (Fig. 2), where *χ*_TIP_ stands for the contribution of temperature-independent paramagnetism.^24^

### Dynamic magnetic properties of CoL4

**Figure 3:**
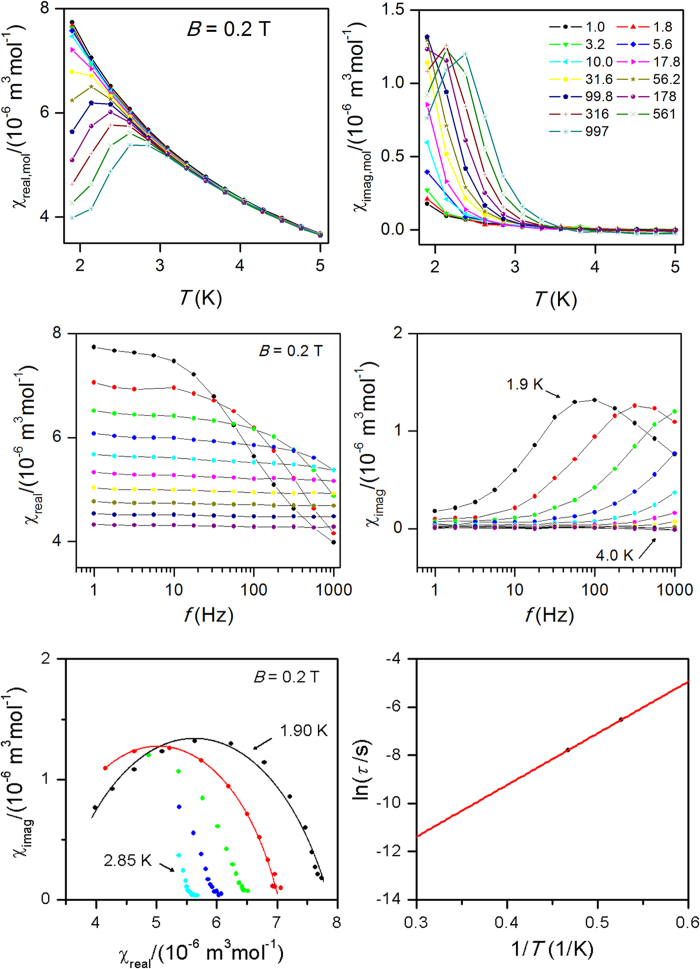
Top: In-phase *χ*_real_ and out-of-phase *χ*_imag_ molar susceptibilities for CoL4 at the applied external field B_dc_ = 0.2 T. Lines serve as guides for the eyes. *Middle*: Frequency dependence of in-phase *χ*_real_ and out-of-phase *χ*_imag_ molar susceptibilities for CoL4 at *B*_dc_ = 0.2 T. Full points – experimental data, full lines – fitted data using equation 7. *Bottom*: Argand (Cole-Cole) plot with full lines showing fitted data using equation 7 (left) and fit of resulting relaxation times according to Arrhenius equation (right).

The negative value of *D*-parameter of CoL4 encouraged us to measure also AC susceptibility data. In zero static magnetic field, there was no out-of-phase susceptibility signal (Figure S1, Supplementary Information), but the field dependent measurement performed at *T* = 1.9 K revealed a slow relaxation of magnetization (Figure S2). Therefore, AC susceptibility measurements were done in non-zero static field, *B*_dc_ = 0.2 T at low temperatures, showing characteristic pattern for slow relaxation of magnetization typically observed for SMM species (Fig. 3). The analysis of susceptibility data for each temperature using the one-component Debye model


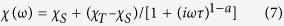


resulted in isothermal (*χ*_T_) and adiabatic (χ_S_) susceptibilities, relaxation times (τ) and distribution parameters (*α*) (Table S2, Supplementary Information). This enabled us to construct the Argand (Cole-Cole) plot (Fig. 3) and using the Arrhenius expression for the temperature dependence of relaxation time resulted in τ_0_ = 1.86⋅10^−8^ s^−1^ and the spin reversal barrier *U* = 21.4 K / 14.7 cm^−1^ (Fig. 3), where only data having maxima in Argand diagram were used. The effective value of *U* is in good agreement with theoretical prediction, *U*_theor_. = |2*D|* *=* 20.1 cm^−1^, based on the single-ion axial zero-field splitting parameter *D* derived from magnetic analysis.

### Preparation and physical characterization of CoL4 : BaFeO mixtures

The solid mixtures of CoL4 and BaFeO were prepared in mass ratios 1:2, 1:1 and 2:1 by ball milling technique. The resulting solids were characterized by a powder X-ray diffraction method in order to prove that the no solid state reaction occurred and that the chemical character of both compounds was preserved (Figure S3, Supplementary Information). Furthermore, also FTIR spectroscopy confirmed that spectra of mixtures are simple sums of individual components spectra (Figure S4). The composition of hybrid material was studied also by UV-VIS spectroscopy. 12 mg of 1:2, 1:1 and 2:1 mixtures were extracted in 40 ml of CHCl_3_ at 40 °C for 20 min in an ultrasonic bath. Extracted solutions were then poured into 100 ml volumetric flasks and additional CHCl_3_ was added to fill the volume up to 100 ml. Appropriate amounts of CoL4 (4 mg for 1:2, 6 mg for 1:1 and 8 mg for 2:1) were dissolved in CHCl_3_, poured into 100 ml volumetric flasks and the volume was filled up to 100 ml. The spectra were measured in the range of 200–1000 nm and compared with the pure phase and mixtures extracts. Using the Beer-Lambert law it was calculated that at least 85% of CoL4 can be extracted back to CHCl_3_ solution from hybrid materials using the above mentioned method (Figure S6) Thus, from the chemical point of view, the resulting mixtures can be considered as two phase systems.

### Static magnetic properties of CoL4 : BaFeO mixtures

**Figure 4:**
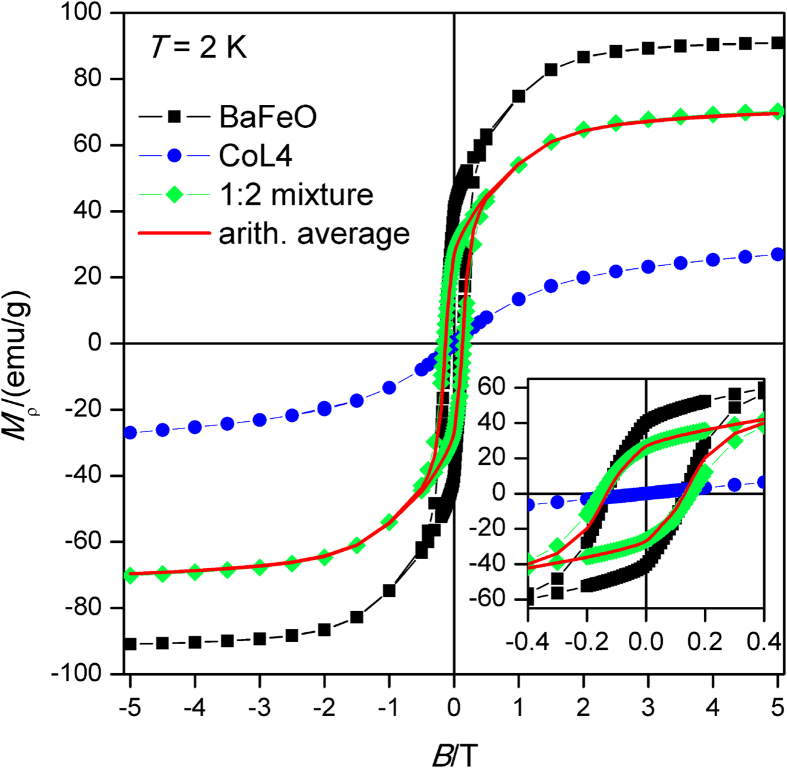
Magnetic hysteresis curves at *T* = *2* K CoL4 (blue circles), BaFeO (black squares), CoL4:BaFeO (1:2) mixture (green diamonds) and the theoretical arithmetic average (1:2) of non-interacting phases (red curve).

In order to eventually identify any magnetic interaction between CoL4 and BaFeO in the studied mixtures, the magnetic hystereses were measured at 2 K in magnetic field range from −5 to +5 T. The commercially available BaFeO behaves as a ferromagnet with saturation magnetization equaled to 90.9 emu/g and coercive field equaled to 0.13 T. On the contrary, CoL4 compound shows no hysteresis at this temperature and magnetization reaches the value of 27.4 emu/g at 5 T. The magnetic properties of 1:2 mixture are shown in Fig. 4, and other mixtures behave in analogous manner. The magnetization of the particular mixture saturates at 70.3 emu/g, which is in good agreement with the calculated arithmetic average equaled to 69.7. Indeed, the calculated curve of arithmetic average coincides with the experimental data very well, there is a slight deviation only at low fields as evident in inset of Fig. 4. This comparison suggests that magnetic interactions between two phases, CoL4 and BaFeO, are either negligible or imperceptible.

### Dynamic magnetic properties of CoL4 : BaFeO mixtures

**Figure 5:**
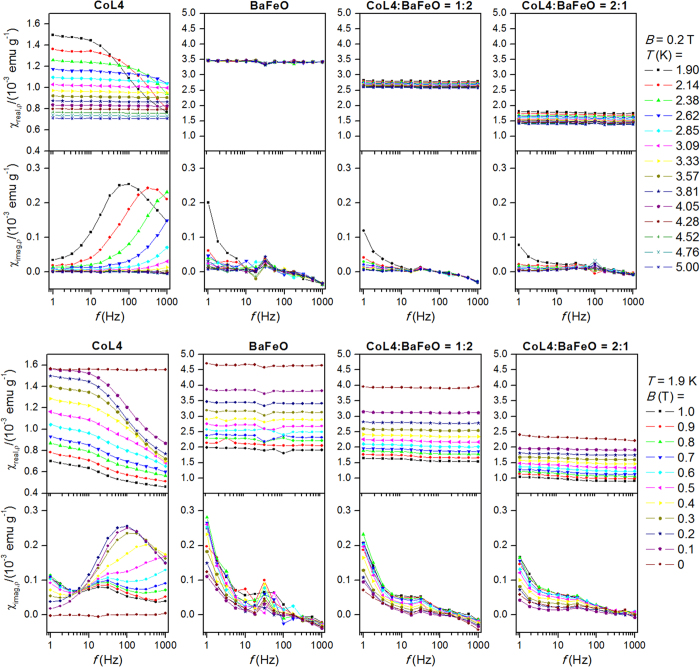
The AC susceptibility data for CoL4, BaFeO, and mixtures of CoL4:BaFeO in the mass ratios of 1:2 and 2:1.

The temperature dependent AC susceptibility data were firstly measured at non-zero (*B*_DC_ = 0.2 T) DC field for all three mixtures. The results for mixtures 1:2 and 2:1 are plotted in Fig. 5, and for mixture 1:1 in Figure S7 (Supplementary Information). Evidently, the characteristic pattern of slow relaxation of magnetization visible both in real and imaginary components of AC susceptibility data of CoL4 is lost in all studied mixtures with BaFeO. The same effect is also manifested in the field depended AC susceptibility data measured at 1.9 K for varying *B*_DC_ from 1.0 to 0.0 T. To ensure that the loss of SRM is not artefact of ball milling, we prepared also the mixture with diamagnetic matrix, Y_2_O_3_, in the mass ratio of 1:2 (Figure S7). In his case, the SRM was clearly observed both in temperature and field dependent AC susceptibility data.

## Discussion

The novel tetracoordinate field-induced single-ion Co(II) molecular magnet was prepared and characterized by various physical techniques resulting in identification of the height of its spin reversal barrier *U* = 21.4 K. The observation of field-induced SRM is inevitably connected to quenching of magnetic tunneling by shifting the energy levels by the Zeeman effect. In CoL4 compound, we observed that the field necessary to do so is relatively small (even below 0.1 T). This property of CoL4 offers an opportunity to test possibility of switching the CoL4 molecules to “field-induced” state simply by using a suitable ferromagnetic substrate. Therefore, the detailed study was performed in order to identify possible magnetic interactions with ferromagnetic barium ferrite (BaFeO). The experimental data proposed that albeit DC magnetic data show imperceptible interference of two mixed solid state phases, the AC susceptibility data surprisingly, but clearly, confirmed suppressing of SRM in CoL4 induced by the ferromagnetic component. It must be noted that the temperature dependent AC study was done in two distinct ways. Firstly, the hybrid samples were magnetized by the static magnetic field (*B* = 2 T) followed by setting zero static magnetic field in no overshoot regime in order to preserve remanent magnetization of BaFeO, and then the dynamic magnetic properties were measured resulting in no out-of-phase signal of CoL4. This motivated us to measure dynamic properties in non-zero static external field in which the pure CoL4 phase possesses maximal out-of-phase signal (*B* = 0.2 T). Again, no out-of-phase signal was observed (Fig. 5). The detailed field dependence AC susceptibility study confirmed that there is no out-of-phase signal of CoL4 in hybrid samples in any static magnetic field up to 1 T (Fig. 5).

The outcome of this work is hard to compare with other systematic works oriented at mutual interaction of SMM and ferro/antiferromagnetic phases, because most of them were done by depositing SMM molecules on various ferro/antiferromagnetic surfaces^26^,^27^,^28^ or diamagnetic substrates^29^,^30^. In such cases, the interactions were dominantly investigated by acquiring hysteresis loops using element-resolved X-ray magnetic circular dichroism (XMCD), while dynamic magnetic information obtainable by AC susceptibility was unreachable. On the contrary, AC susceptibility measurements were successfully employed in SMM molecules attached on gold nanoparticles^31^,^32^. However, all the above mentioned methods did not preserve original crystal structure of SMM and therefore the molecular geometry is usually affected by processing the hybrid structures. In order to avoid this drawback, as the magnetic anisotropy of 3*d* metal complexes is significantly affected by change in their molecular geometry, we directly used the crystalline materials of CoL4 and BaFeO in the preparation of the studied mixtures.

To summarize, this article reports on magnetic properties of a novel tetracoordinate field-induced single-molecule magnet CoL4. We studied its magnetic properties and we used it as a constituent for the preparation of mixtures with ferromagnetic barium ferrite (BaFeO). It was observed that suppressing of slow-relaxation of magnetization (SRM) on the complex molecule occurred in these materials by the interaction with barium ferrite. On the contrary, CoL4 in the mixture prepared from CoL4 and diamagnetic Y_2_O_3_ preserves slow relaxation of magnetization as was observed in pure CoL4, which indirectly shows that the suppression of SRM in CoL4-BaFeO hybrid materials has origin in BaFeO ferromagnetic component.

## Methods

### Materials

All solvents and other chemicals were purchased from commercial sources (Sigma Aldrich) and used as received. BaFe_12_O_19_ was supplied from Aldrich co. (CAS 11138-11-7), herein abbreviated as BaFeO.

### Synthesis of CoL4

0.13 g of CoCl_2_ (1 mmol) was dissolved in 20 cm^3^ of hot methanol and then, 0.20 g of KNCS (2 mmol) was added with stirring. The color of the solution turned violet immediately. After 10 min of stirring and heating, 0.24 g of benzimidazole (2 mmol) was added to the reaction mixture and resulting blue solution was refluxed for next 10 min. Then, the solution was cooled down and solid KCl was filtered off through a paper filter. Resulting solution was left to evaporate slowly and after several days blue microcrystalline powder appeared. Single crystals can be obtained by layering of the methanol solution of CoL4 by *n*-hexane, or by slow diffusion of diethyl ether to such solution. Yield: 74%., Anal. Calcd for C_16_H_12_Co1N_6_S_2_ (*M*_r _= 411.37) (%), C, 46.7, H, 2.94, N, 20.43, Found: C, 46.6, H, 2.91, N, 20.37. IR mid (cm^−1^): ν(N–H) = 3283 (m), ν(C–H)_arom_ = 3113 (w), ν(NCS) = 2072 (s).

### Synthesis of CoL4 : BaFeO mixtures

40 mg of CoL4 and 40 mg of BaFeO were weighted out and transferred into small ball mill (Dentsply Rinn, model 3110) and milled for 15 minutes in order to prepare a mixture with the mass ratio of 1: 1. The other mixtures were prepared analogously using the appropriate mass ratios of 1: 2 and 2 : 1.

### Crystallography

Single crystal X-ray diffraction data were collected using Oxford diffraction Xcalibur2 CCD diffractometer with a Sapphire CCD detector (Mo-Kα radiation, λ = 0.71073 Å). The structure was solved by direct methods using SHELXS97^33^ incorporated into the WinGX program package^34^. The structure was refined using full-matrix least-squares on *F*_o_^2^ − *F*_c_^2^ with SHELXL-97^33^ with anisotropic displacement parameters for non-hydrogen atoms. All the hydrogen atoms were found in differential Fourier maps and their parameters were refined using a riding model with *U*_iso_(H) = 1.2 *U*_eq_ (atom of attachment). The crystal structure was visualized using the Mercury software^35^. Crystal data, for CoL4: *P*-1, *a* = 8.6216(4) Å, *b* = 9.3539(4) Å, *c* = 12.7307(6) Å, *α* = 75.218(4) °, *β* = 81.213(4) °, *γ* = 63.683(4) °, V = 888.88(7) Å^3^, *Z*`= 2, *ρ* = 1.537 g.cm^−1^, *μ* = 1.211 mm^−1^, parameters/ restraints/unique reflextions = 226/0/2249, *R*_int_ = 0.0214, *R*_1_/*w*R_2_(*I* > 2*σ*(I)) = 0.0447/0.0983, *R*_1_/*w*R_2_(all data) = 0.0831/0.1198; CCDC no. 1040575.

### Physical methods

Elemental analysis (C, H, N) was performed on a Flash 2000 CHNO-S Analyzer (Thermo Scientific). Infrared (IR) spectra of the complexes were recorded on a Thermo Nicolet NEXUS 670 FT-IR spectrometer (Thermo Nicolet) employing the ATR technique on a diamond plate in the range of 400–4000 cm^–1^. Temperature dependence of the magnetization at *B* = 0.1 T from 1.9 to 300 K and the isothermal magnetizations at *T* = 2.0 and 5.0 K up to *B* = 5 T were measured using MPMS XL-7 SQUID magnetometer (Quantum Design). The experimental data were corrected for diamagnetism. Measurements of AC susceptibility were carried out in a 3.8 Oe ac field oscillating at various frequencies from 1 to 1000 Hz and with various dc fields. SEM images and energy-dispersive X-ray (EDX) spectroscopy data were recorded on a Hitachi 6600 FEG microscope. Powder samples were placed on an aluminum holder with double-sided adhesive carbon tape. The accelerating voltages used were in the range of 5−15 keV. The X-ray powder diffraction patterns of all solid samples were recorded on an MiniFlex600 (Rigaku) instrument equipped with the Bragg−Brentano geometry, and with iron-filtered Cu Kα_1,2_ radiation.

### *Ab initio* calculations

All *ab initio* calculations were performed with ORCA 3.0.1 computational package^36^ on the experimental X-ray structure of CoL4, without employing optimization of the molecular structure by computational methods. The relativistic effects were also included in the calculation with zero order regular approximation (ZORA)^37^,^38^ together with the scalar relativistic contracted version of def2-TZVP(-f) basis functions.^39^ The DFT calculations were based on B3LYP functional^40^,^41^,^42^,^43^ and utilized the RI approximation with the decontracted auxiliary def2-TZV/J Coulomb fitting basis set and the chain-of-spheres (RIJCOSX) approximation to exact exchange^44^. Increased integration grids (Grid5 and GridX5in ORCA convention) and tight SCF convergence criteria were used also.

The calculations of ZFS parameters were based on state average complete active space self-consistent field (SA-CASSCF)^45^ wave functions complemented by N-electron valence second order perturbation theory (NEVPT2)^46^,^47^,^48^,^49^,^50^. The active space of the CASSCF calculations comprised of seven electrons in five metal-based *d*-orbitals (CAS(7,5)). The state averaged approach was used, in which all ten quartet states and forty doublets states were equally weighted. The calculations utilized the RI approximation with the decontracted auxiliary def2-TZV/C and def2-SVP/C Coulomb fitting basis sets and the chain-of-spheres (RIJCOSX) approximation to exact exchange. Increased integration grids (Grid4 in ORCA convention) and tight SCF convergence criteria were used. The ZFS parameters, based on dominant spin−orbit coupling contributions from excited states, were calculated through quasi-degenerate perturbation theory (QDPT),^51^ in which an approximations to the Breit-Pauli form of the spin-orbit coupling operator (SOMF approximation)^52^ and the effective Hamiltonian theory^53^ were utilized.

## Additional Information

**How to cite this article**: Nemec, I. *et al*. Suppressing of slow magnetic relaxation in tetracoordinate Co(II) field-induced single-molecule magnet in hybrid material with ferromagnetic barium ferrite. *Sci. Rep*. **5**, 10761; doi: 10.1038/srep10761 (2015).

## Supplementary Material

Supplementary Information

## Figures and Tables

**Table 1:** Selected structural parameters for tetracoordinate [Co(L)_2_(A)_2_] compounds, (L = monodentate ligand, A = anionic ligand). Abbreviations: qu = quinoline, pic = *γ*-picoline, PPh_3_ = triphenylphosphane.

compound	chromophore	Co-L/Å	Co-A/Å	*δ*/°	*Δ*/°	*D*/cm^−1^	ref.
[Co(NCS)_2_(qu)_2_]	{CoN_2_N’_2_}	2.036	1.937	+6.2	24.4	+6.2	14
[CoCl_2_(qu)_2_]	{CoN_2_Cl_2_}	2.070	2.244	−1.6	32.4	−5.2	15
[CoCl_2_(pic)_2_]	{CoN_2_Cl_2_}	2.051	2.233	−9.8	24.3	−5.3	16
[CoCl_2_(PPh_3_)_2_]	{CoP_2_Cl_2_}	2.383	2.212	−14.1	27.5	−11.8	17
[CoCl_2_(PPh_3_)_2_]	{CoP_2_Br_2_}	2.385	2.349	−13.6	27.0	−13.0	17
CoL4	{CoN_2_N’_2_}	1.991	1.940	−6.3	22.3	−10.1	this work
