# Supplementary material for: Suppressing of slow magnetic relaxation in tetracoordinate Co(II) field-induced single-molecule magnet in hybrid material with ferromagnetic barium ferrite
Source: Sci Rep. 2015 Jun 3;5:10761. doi: 10.1038/srep10761 (PMC4454148; doi:10.1038/srep10761)
Supplement: Supplementary Information [file srep10761-s1.doc]

**Supplementary information**

Suppressing of slow magnetic relaxation in tetracoordinate Co(II) field-induced single-molecule magnet in hybrid material with ferromagnetic barium ferrite

Ivan Nemec, Radovan Herchel, Zdeněk Trávníček^*^

*Department of Inorganic Chemistry, Regional Centre of Advanced Technologies and Materials, Faculty of Science, Palacký University, 17. listopadu 12, CZ-771 46 Olomouc, Czech Republic.*

*^*^ E-mail:* [*zdenek.travnicek@upol.cz*](mailto:zdenek.travnicek@upol.cz)


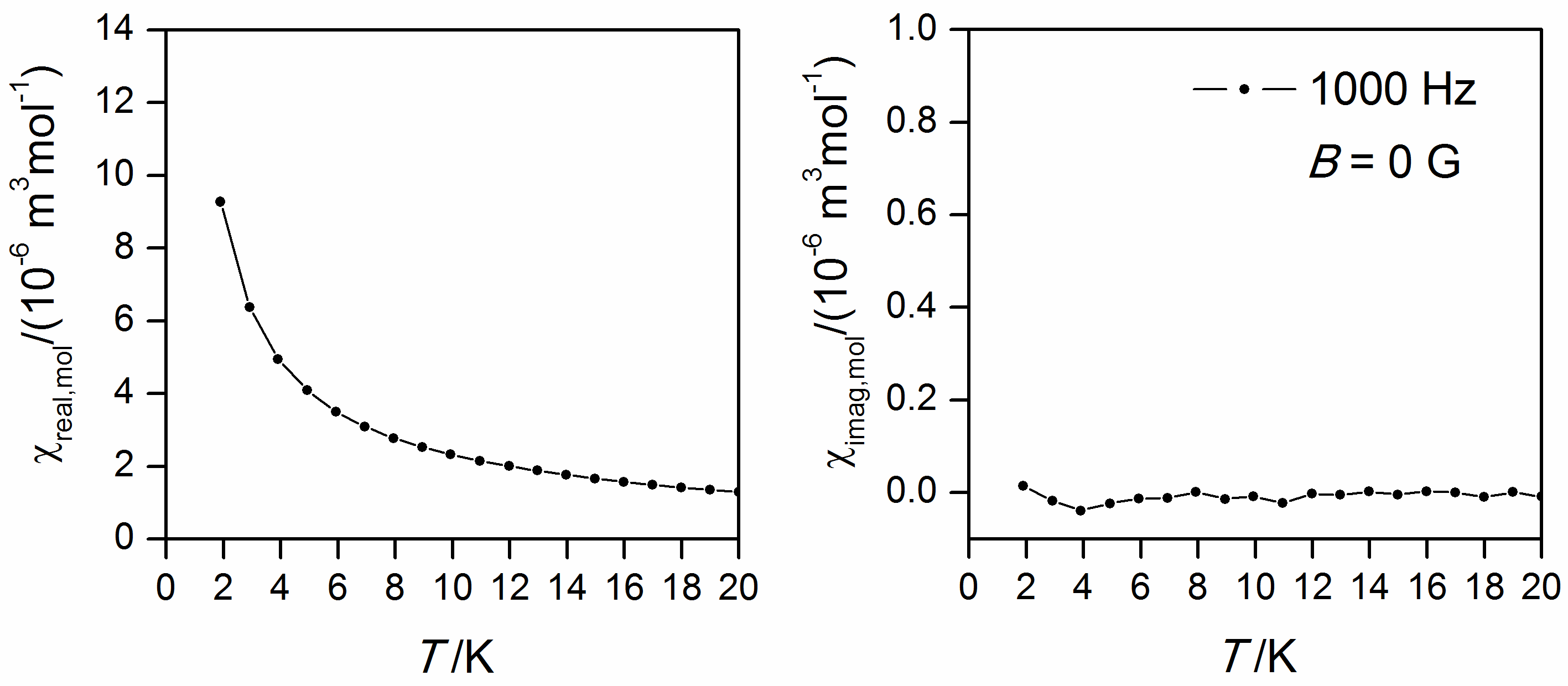


**Figure S1**. In-phase *χ*_real_ and out-of-phase *χ*_imag_ molar susceptibilities for CoL4 at zero external field *B*_dc_ = 0.0 T. Lines serve as guides for the eyes.


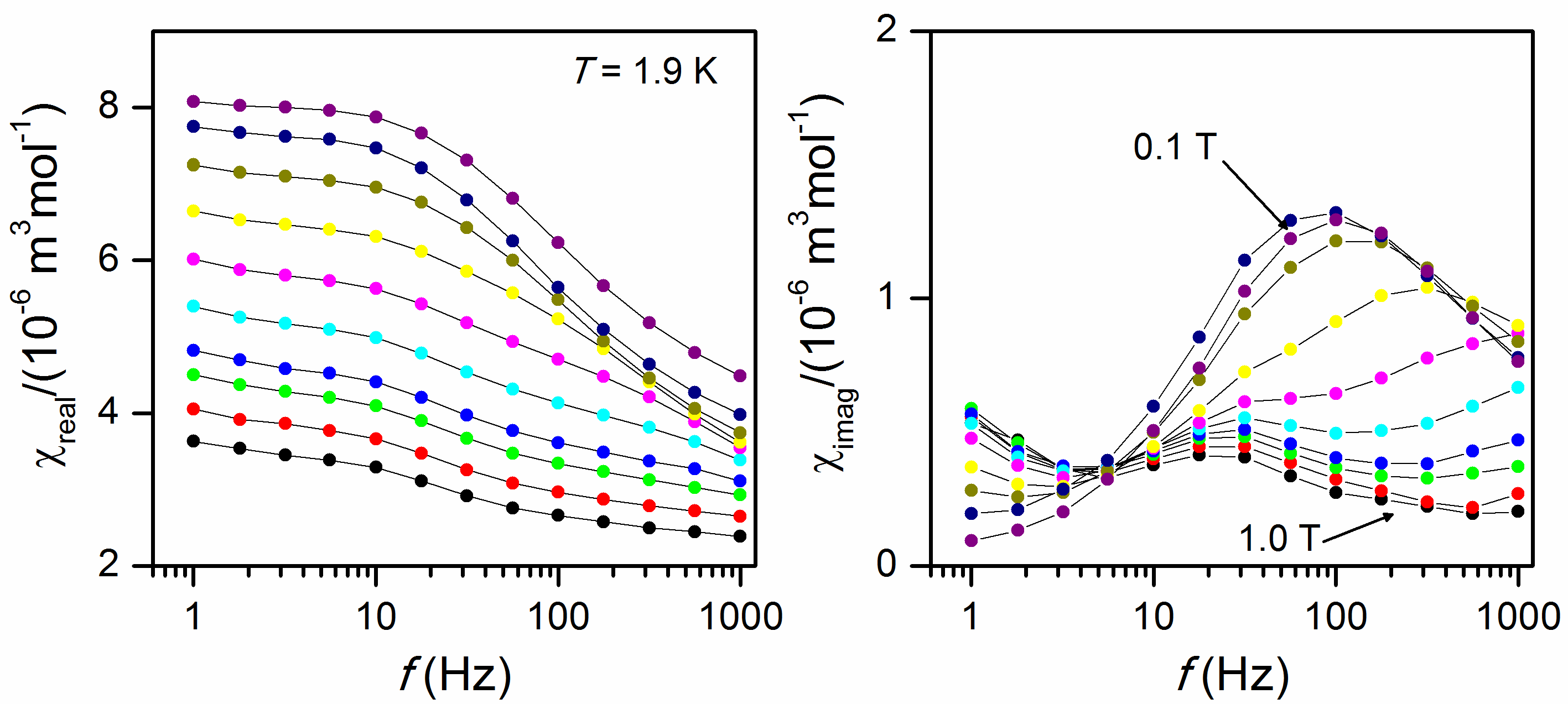


**Figure S2**. Frequency dependence of in-phase *χ*_real_ and out-of-phase *χ*_imag_ molar susceptibilities for CoL4 at *T* = 1.9 K for various static magnetic fields *B*_dc_ = 0.1-1.0 T. Lines serve as guides for the eyes.

**Figure S3**. PXRD of pure phases and CoL4:BaFeO mixtures.


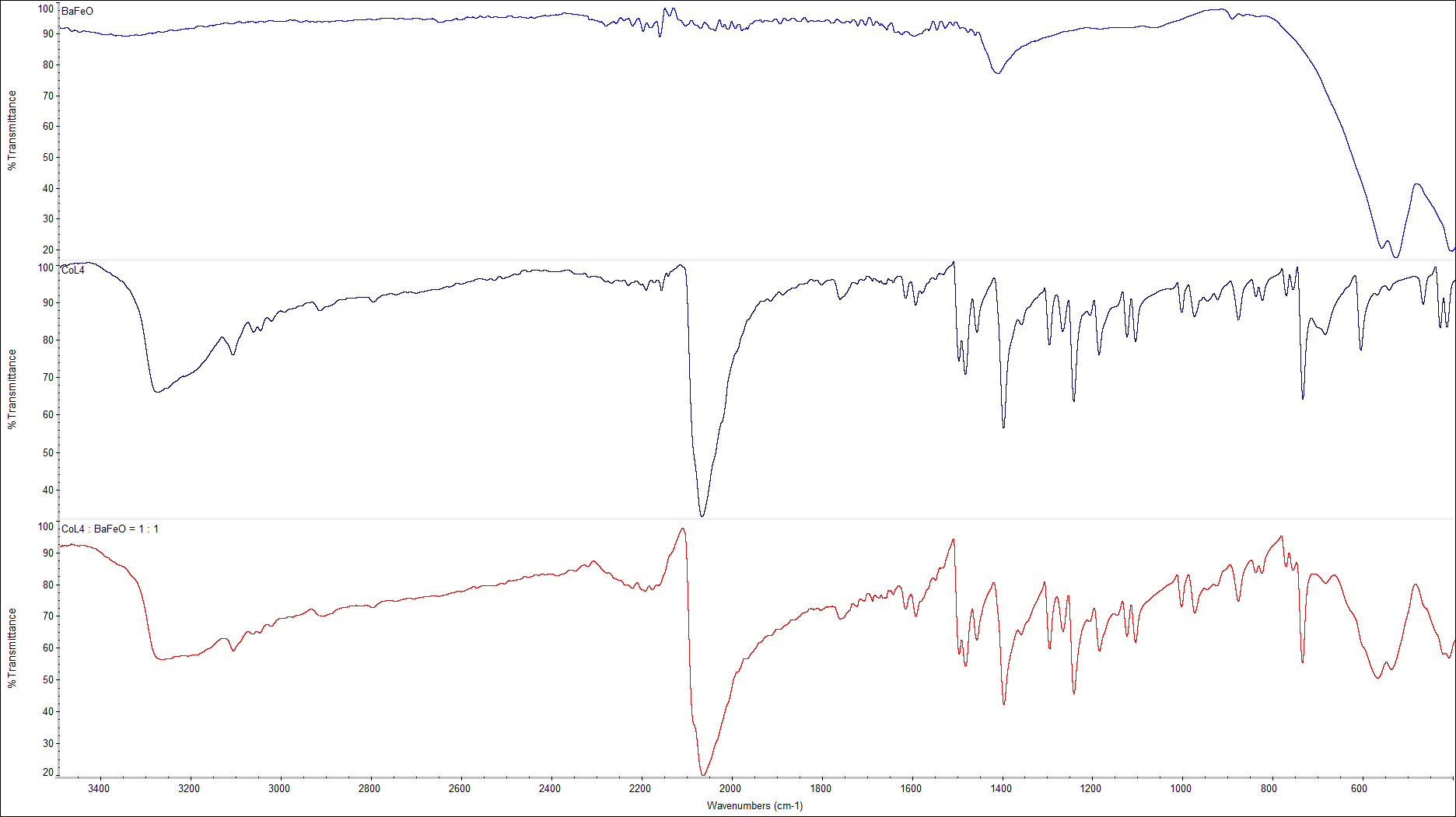


**Figure S4**. FTIR spectra of PXRD of BaFeO (top), CoL4 (bottom) and 1:1 mixture of CoL4 and BaFeO (middle).

| 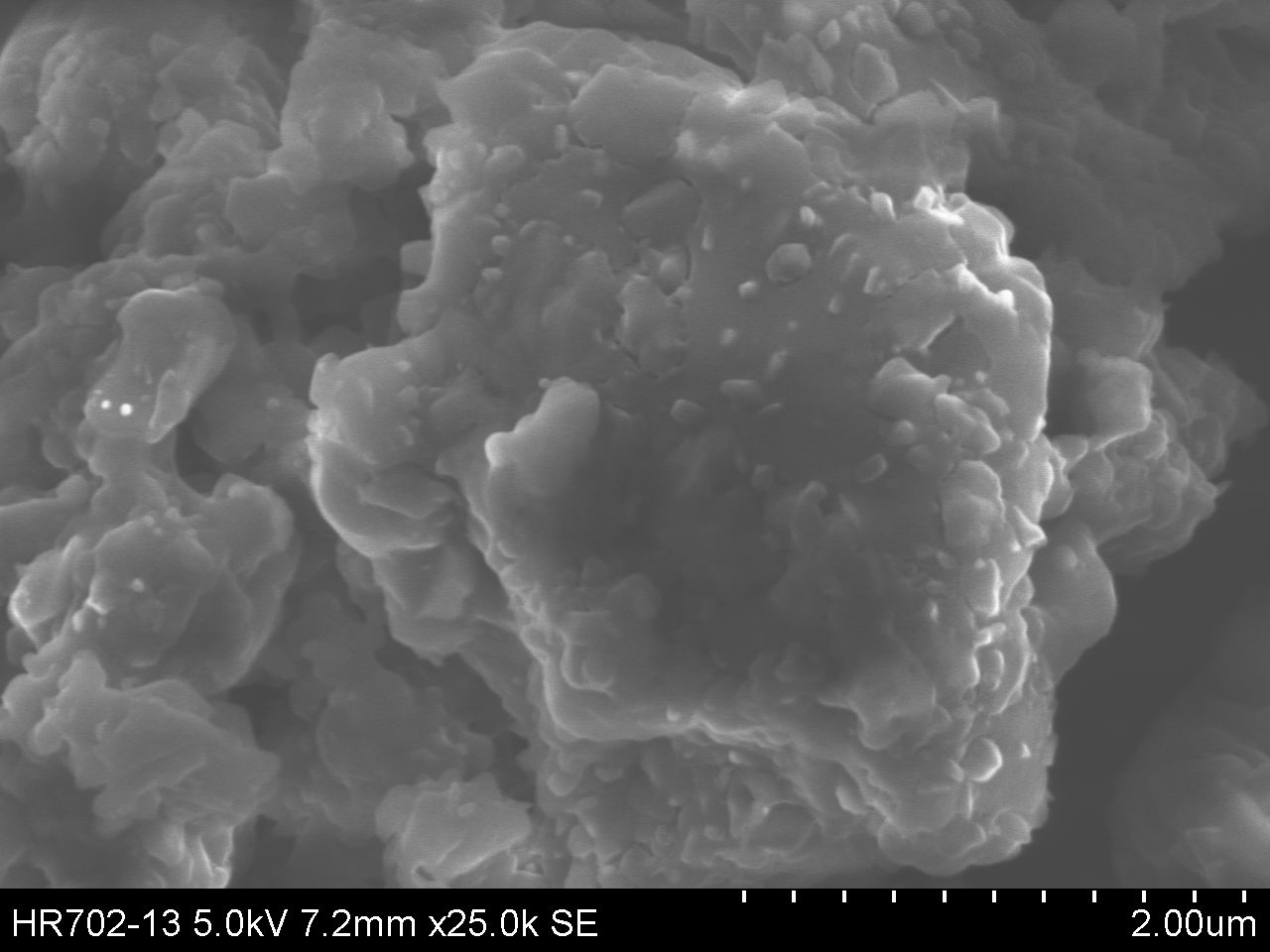 | 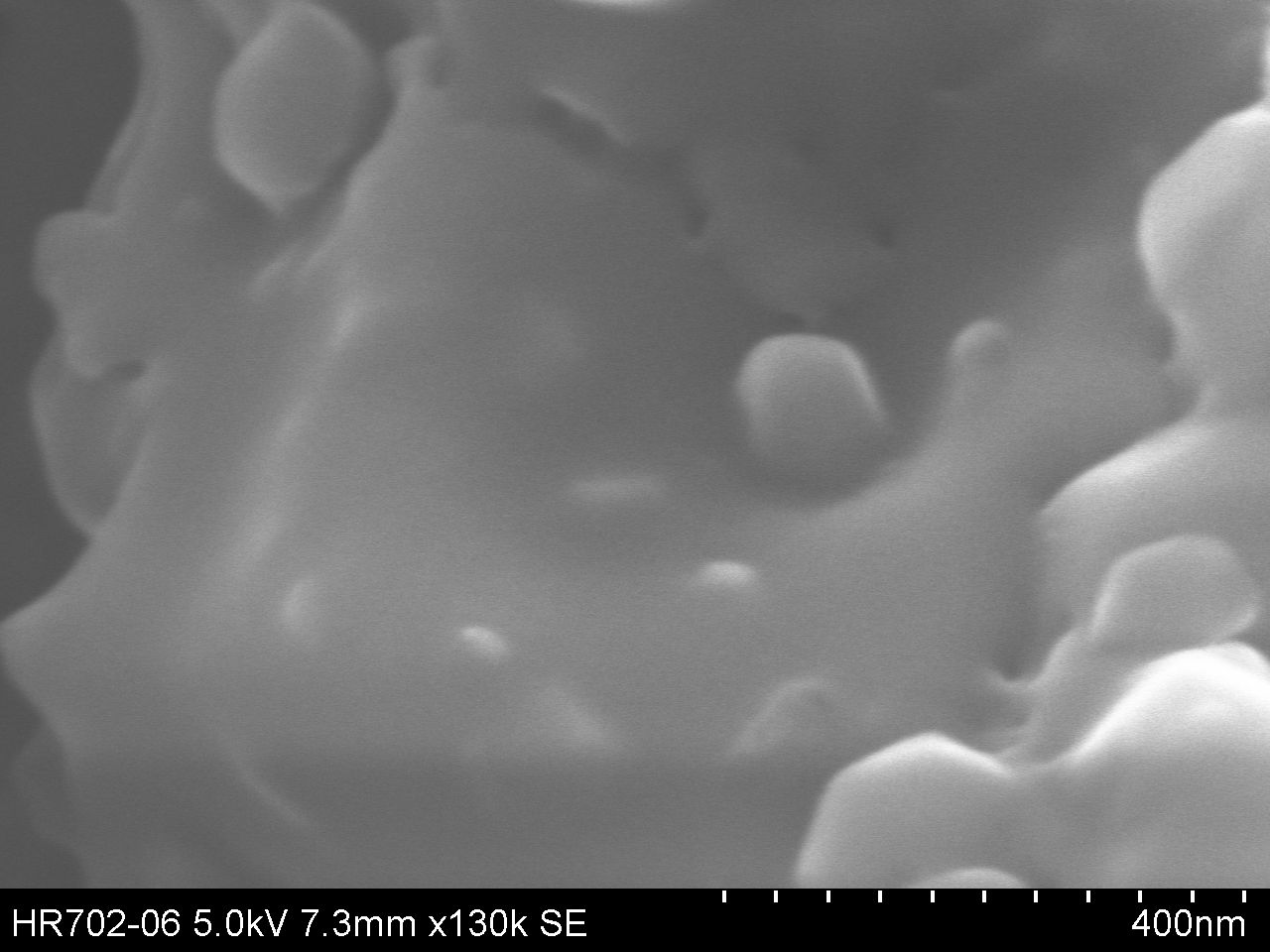 |
| --- | --- |
| 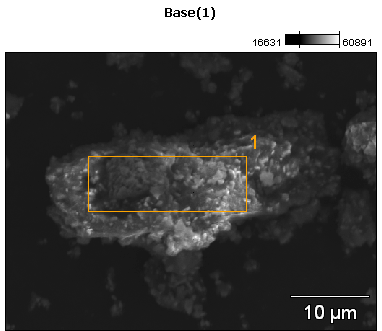 | 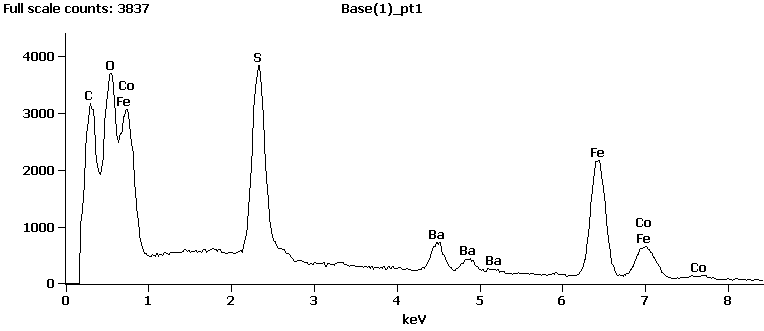 |

**Figure S5**. SEM/EDS spectra of the CoL4 : BaFeO mixture 1 : 1.


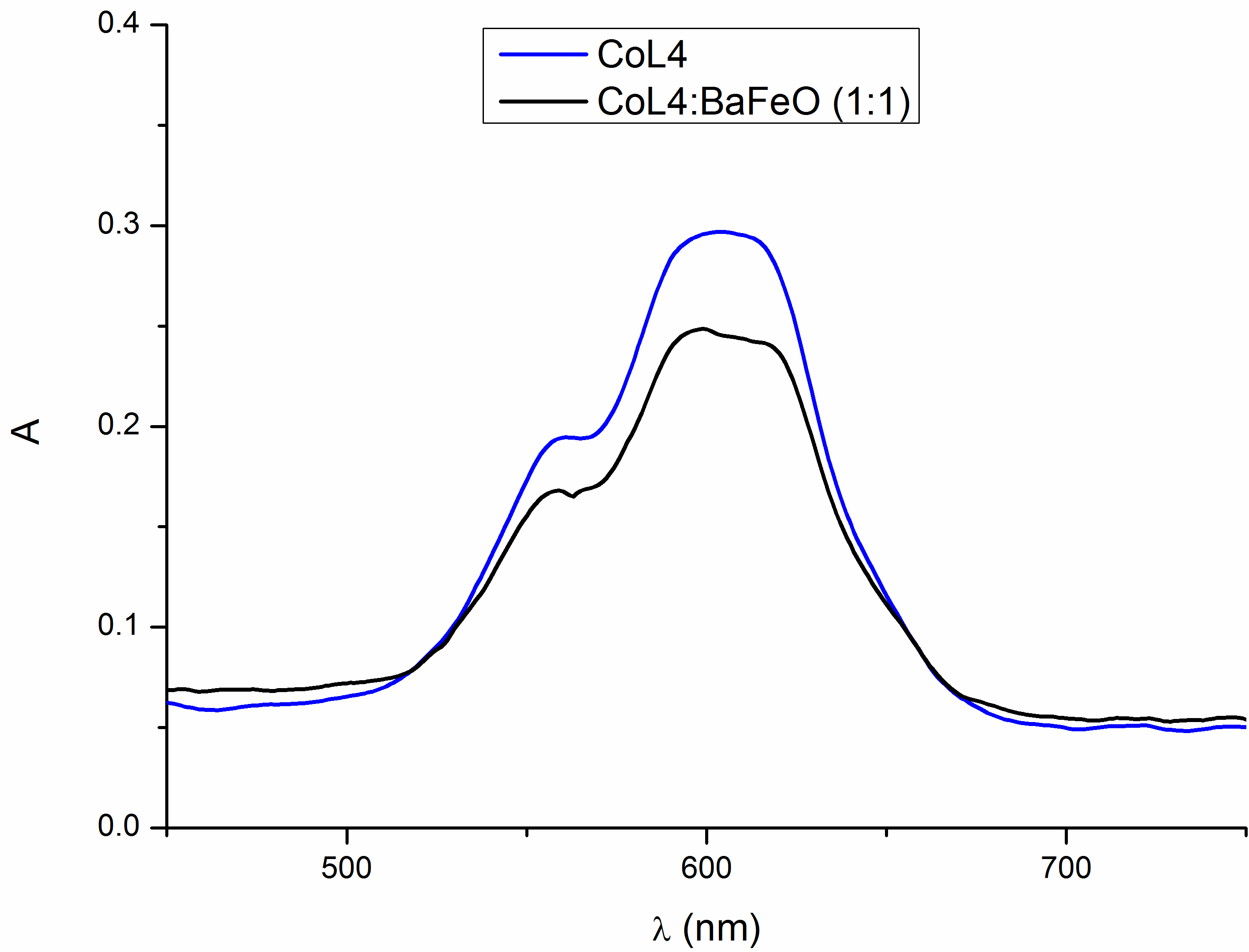


**Figure S6**. UV/Vis spectra of pure CoL4 (blue) and CoL4 extracted from the CoL4:BaFeO mixture (1:1, black).


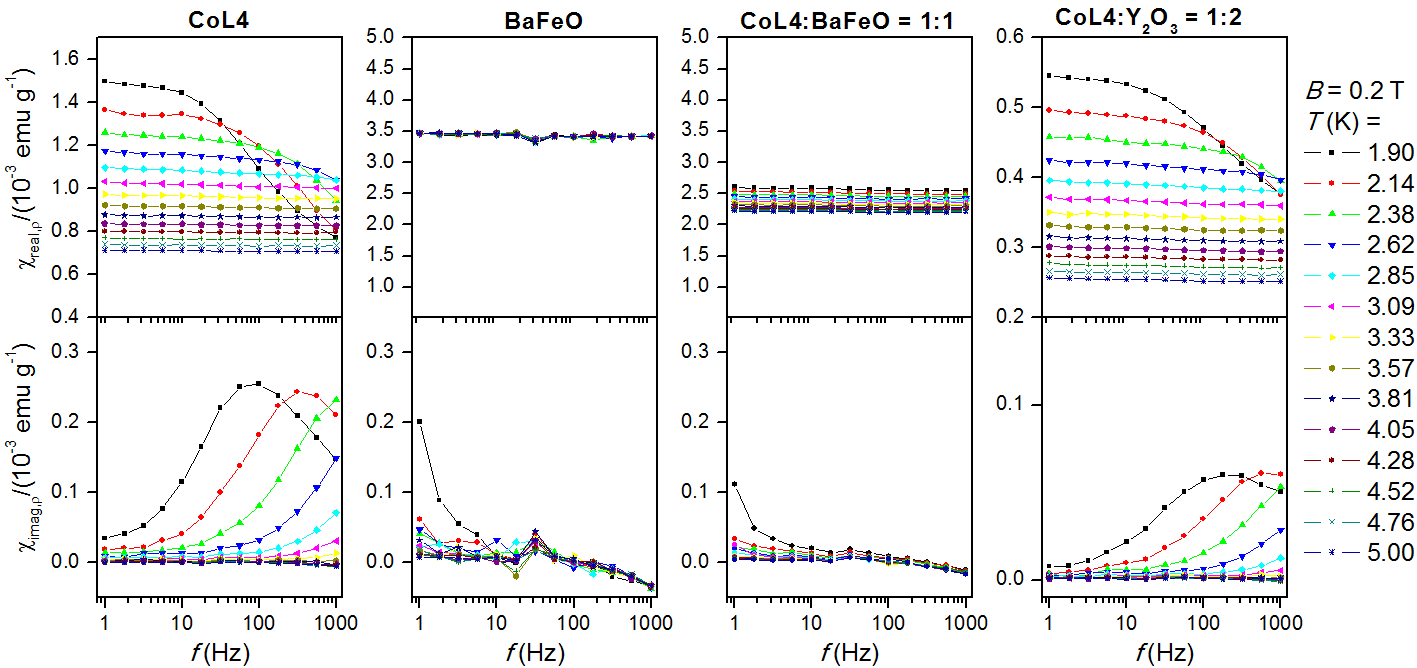


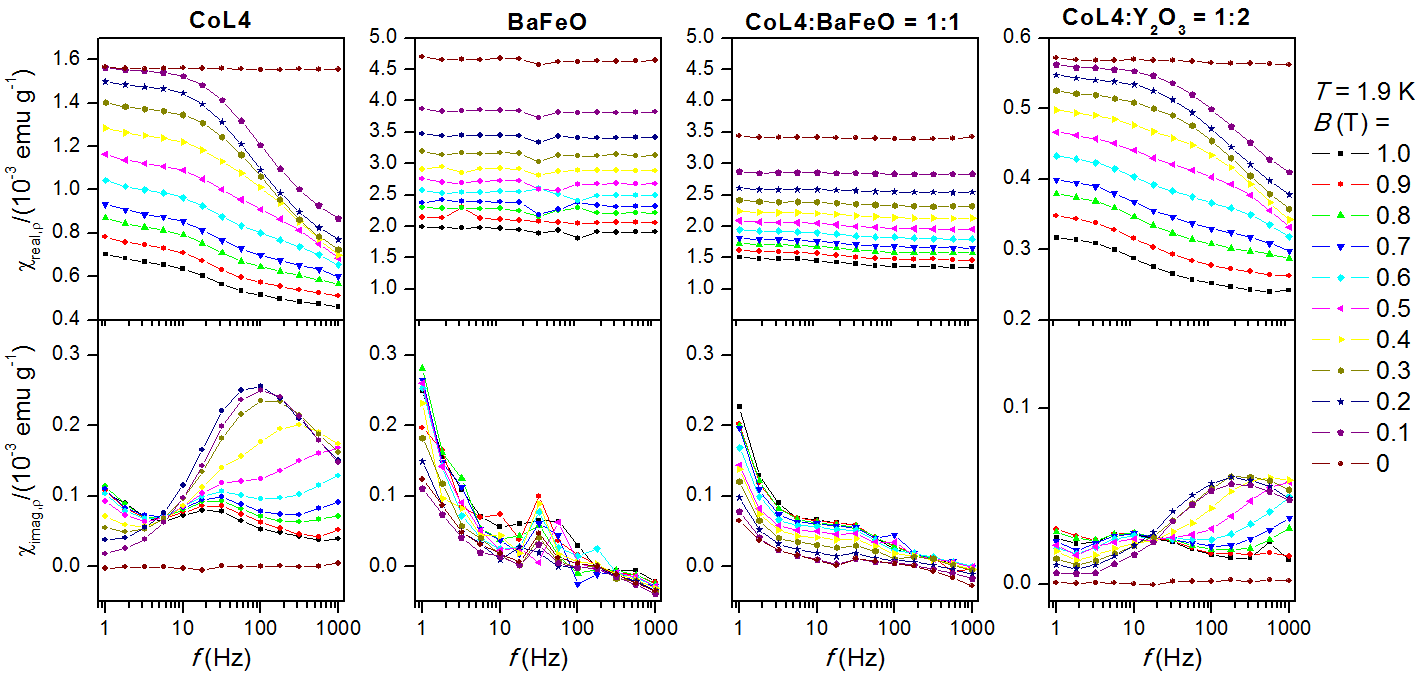


**Figure S7**. The AC susceptibility data for CoL4, BaFeO, and mixtures of CoL4 : BaFeO (1 : 1) and CoL4 : Y_2_O_3_ (1 : 2).

**Table S1.** Individual contributions to *D*-tensor for molecular fragment of CoL4 calculated by CASSCF/NEVPT2.

| Multiplicity | Root | *D* | *E* |
| --- | --- | --- | --- |
| 4 | 0 | 0 | 0 |
| 4 | 1 | -25.962 | 0.704 |
| 4 | 2 | 8.281 | -5.451 |
| 4 | 3 | 6.181 | 4.122 |
| 4 | 4 | -0.173 | 0.084 |
| 4 | 5 | -0.009 | 0.012 |
| 4 | 6 | 0.029 | 0.019 |
| 4 | 7 | 0.001 | 0.001 |
| 4 | 8 | 0 | 0 |
| 4 | 9 | -0.003 | 0 |
| 2 | 0 | -0.222 | -0.201 |
| 2 | 1 | -0.13 | 0.01 |
| 2 | 2 | -0.027 | 0.023 |
| 2 | 3 | -0.016 | 0.048 |
| 2 | 4 | 0.065 | -0.028 |
| 2 | 5 | 0.001 | 0 |
| 2 | 6 | 4.856 | -0.414 |
| 2 | 7 | -2.113 | -1.807 |
| 2 | 8 | -2.522 | 2.197 |
| 2 | 9 | 0.122 | -0.138 |
| 2 | 10 | 0.62 | -0.004 |
| 2 | 11 | -0.022 | 0.013 |
| 2 | 12 | 0.019 | -0.002 |
| 2 | 13 | -0.021 | -0.002 |
| 2 | 14 | -0.076 | -0.051 |
| 2 | 15 | -0.162 | 0.161 |
| 2 | 16 | 0.026 | -0.005 |
| 2 | 17 | -0.005 | 0.016 |
| 2 | 18 | -0.003 | -0.002 |
| 2 | 19 | 0.017 | -0.005 |
| 2 | 20 | -0.004 | -0.001 |
| 2 | 21 | 0 | 0 |
| 2 | 22 | -0.57 | -0.597 |
| 2 | 23 | -0.008 | -0.006 |
| 2 | 24 | -0.527 | 0.527 |
| 2 | 25 | 0.51 | 0 |
| 2 | 26 | -0.002 | 0.002 |
| 2 | 27 | -0.001 | 0.001 |
| 2 | 28 | 0 | 0 |
| 2 | 29 | 0.191 | -0.023 |
| 2 | 30 | -0.01 | -0.073 |
| 2 | 31 | -0.105 | 0.099 |
| 2 | 32 | 0.001 | -0.005 |
| 2 | 33 | -0.003 | 0.002 |
| 2 | 34 | 0.001 | 0 |
| 2 | 35 | -0.001 | 0.001 |
| 2 | 36 | -0.009 | 0.008 |
| 2 | 37 | -0.003 | -0.008 |
| 2 | 38 | -0.011 | 0.009 |
| 2 | 39 | 0.013 | -0.013 |

**Table S2.** Parameters of one-component Debye model for **CoL4** derived according Eq.7 in main text.

| *T*/K | *χ*_S_/(10^-6^ m^3^mol^-1^) | *χ*_T_/(10^-6^ m^3^mol^-1^) | α | *τ*/(10^-3^ s) |
| --- | --- | --- | --- | --- |
| 1.90 | 3.407 | 7.867 | 0.312 | 1.479 |
| 2.14 | 3.043 | 7.032 | 0.276 | 0.4172 |
